# Obesity: Childhood BMI Rises with Prenatal Exposure to Hexachlorobenzene

**Published:** 2008-12

**Authors:** David J. Tenenbaum

A prospective study published in the October 2008 issue of *Acta Paediatrica* has found an association between prenatal exposure to hexachlorobenzene (HCB) and being overweight or obese in childhood. The study was the first prospective look at prenatal exposure of HCB in a general population, says corresponding author Agnes Smink, now a research coordinator at consultancy SHL Group–Breda. The study relied on body mass index (BMI), a common gauge of overweight and obesity.

HCB is one of many organochlorine compounds restricted or banned by the Stockholm Convention on Persistent Organic Pollutants, which was signed in 2001. The compound was widely used, starting in 1933, to kill fungi on crop seeds and in some manufacturing processes. Its commercial use in the United States ceased in 1965. HCB bioaccumulates in fat and breaks down slowly.

In humans, prenatal exposure to HCB has been shown to affect age at menarche and menopause, asthma, and fertility, as well as increase the relative risk of attention deficit/hyperactivity disorder. Other organochlorine compounds, including DDT and the polychlorinated biphenyls (PCBs), have also been implicated in causing damage *in utero*.

In 1997, the research group began recruiting expectant mothers on the Spanish Mediterranean island of Minorca. They measured HCBs, PCBs, the pesticide DDT, and the DDT metabolite DDE in umbilical cord blood of 405 children. The children’s height and weight was measured at birth and at age 6.5 years.

Analysis showed that each doubling in cord blood HCB levels was associated with a weight increase of 1.14 kg (2.5 lb), but the children’s height did not differ significantly. The relative risk of being overweight was a statistically significant 1.7 times higher per doubling in cord blood HCB level, and the relative risk for obesity was a nonsignificant 2.0 times higher. The association between HCB concentration and elevated BMI was independent of maternal socioeconomic status, weight, or education, or child’s birth order or birth weight. There was no observed correlation between elevated BMI and PCBs, DDT, or DDE.

“This study provides an important piece of evidence that obesity may be related not only to junk food and lack of exercise but also to halogenated compounds or endocrine disruptors,” says Wilfried Karmaus, a professor of epidemiology at the University of South Carolina. In his study of Michigan women who ate large quantities of home-caught fish, maternal levels of DDE did correlate with BMI and weight in the women’s adult children aged 20 to 50. That study, which relied on historic analyses of blood samples in which HCB had not been measured, will be published in a forthcoming issue of *Occupational and Environmental Medicine*.

The fact that different studies find different correlations between organochlorines and childhood obesity is troubling, Karmaus admits. “It bothers me a little bit that the Spanish found [a link with] HCB and we found [one with] DDE,” he says. “But they are both endocrine disruptors, and they may work on a comparable mechanism. There is some indication that these substances may disrupt normal development or the metabolism of fat, but we don’t know which substance and which mechanism.”

Smink cautions that the new study does not suggest that HCB, or indeed any other organochlorine, is the sole explanation for overweight and obesity, which are estimated by the World Health Organization to affect at least 1 billion and 300 million people, respectively, worldwide. “It is important to keep in mind that a lot of factors are involved in the obesity epidemic,” she says. “I think that the proportion of the ongoing obesity epidemic is minimal when it comes to HCB exposure. However, it is important to reduce prenatal exposure to toxicants like HCB to prevent health problems like overweight. It is not clear if the observed effects on body weight remain after the age of six years.”

Because most HCB exposure comes through diet, especially meat, “a mother can prevent exposure by paying attention to where the local pollution sources are located and avoid eating food which has been grown there,” Smink says.

“Pregnancy is a really important time for development,” says Karmaus. “During pregnancy, you are exposed to [all the toxicants] your mother has collected in twenty or thirty years.” Prevention is key, he says, because “as a fetus you can't decide not to live under these conditions.”

## Figures and Tables

**Figure:**
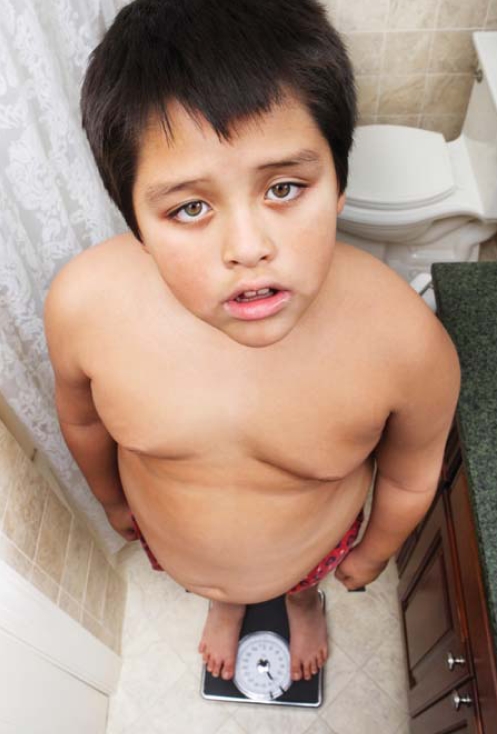
Child obesity may begin with prenatal exposures.

